# How to demonstrate the impact of ivabradine on suppressing ventricular arrhythmia

**DOI:** 10.1590/1806-9282.20240093

**Published:** 2024-05-17

**Authors:** Daichi Sannomiya, Naoya Kataoka, Teruhiko Imamura

**Affiliations:** 1 University of Toyama, Second Department of Internal Medicine – Toyama, Japan.

Dear Editor,

Ivabradine, an inhibitor of the I_f_ current predominantly expressed in the sinoatrial node, improves mortality and morbidity in patients with heart failure and reduced ejection fraction (HFrEF) as well as sinus rhythm by modulating heart rate^1^. However, the impact of ivabradine on reducing the burden of ventricular arrhythmia remains unknown. Pay and colleagues demonstrated that ivabradine therapy was associated with a lower incidence of appropriate implantable cardioverter-defibrillator discharge but was not associated with mortality in the HFrEF cohort^2^. Several concerns have been raised.

The cornerstone randomized controlled study, the SHIFT trial, demonstrated the impact of ivabradine on reducing cardiovascular death or heart failure readmission in the HFrEF cohort^1^. Could the authors explain the reason for the discrepancy between the two studies? In the SHIFT trial, patients with left ventricular ejection fraction <35%, heart rate ≥70 bpm, and sinus rhythm were included and received ivabradine^1^. How many patients in the authors’ study satisfy the same inclusion criteria? In the SHIFT trial, the dose of ivabradine was up-titrated to achieve a heart rate between 50 and 60 bpm^1^. Could patients achieve sufficient heart rate reduction during ivabradine therapy? For the achievement of heart rate optimization, our team recently proposed to minimize the overlap between the E-wave and A-wave in the Doppler echocardiographic transmitral flow during ivabradine therapy to maximize cardiac output (echo-guided heart rate modulation therapy)^3^.

The mechanism of why ivabradine suppresses ventricular arrhythmia remains uncertain. Another heart failure medication, sacubitril/valsartan, suppresses the incidence of ventricular arrhythmia by facilitating cardiac reverse remodeling^4^. Did the authors have successive echocardiographic data during ivabradine therapy? Ivabradine has the potential to stabilize hemodynamics and give us a chance to up-titrate the dose of beta-blockers, which may assist in suppressing ventricular arrhythmia. Were heart failure medications up-titrated during ivabradine therapy?

The reason for implantable cardioverter-defibrillator implantation is unclear^2^. The incidence of ventricular arrhythmia should be higher in patients who received the devices for secondary prevention versus primary prevention.
